# Regional cortical thinning of the orbitofrontal cortex in medication-naïve female patients with major depressive disorder is not associated with MAOA-uVNTR polymorphism

**DOI:** 10.1186/s12991-016-0116-0

**Published:** 2016-10-12

**Authors:** Eunsoo Won, Sunyoung Choi, June Kang, Min-Soo Lee, Byung-Joo Ham

**Affiliations:** 1 Department of Psychiatry, Korea University Anam Hospital, Korea University College of Medicine, 73, Inchon-ro, Seongbuk-gu, Seoul, 136-705 Republic of Korea; 2 Department of Brain and Cognitive Engineering, Korea University, Seoul, Republic of Korea; 3 Department of Biomedical Science, Korea University, Seoul, Republic of Korea; 4 Department of Psychiatry, Korea University Anam Hospital, Korea University College of Medicine, Seoul, Republic of Korea

**Keywords:** Major depressive disorder, Orbitofrontal cortex thickness, Monoamine oxidase A-upstream variable number of tandem repeats

## Abstract

**Background:**

Orbitofrontal cortex alterations have been suggested to underlie the impaired mood regulation in depression. *MAOA*-uVNTR (monoamine oxidase A-upstream variable number of tandem repeats) polymorphism has been reported to be associated with major depressive disorder by various studies. The influence of *MAOA*-uVNTR genotype on function and structure of the orbitofrontal cortex has previously been reported. In this study, we investigated the difference in orbitofrontal cortex thickness between medication-naïve female patients with major depressive disorder and healthy controls, and the influence of *MAOA*-uVNTR genotype on orbitofrontal cortex thickness in depression.

**Methods:**

Thirty-one patients with major depressive disorder and 43 healthy controls were included. All participants were subjected to T1-weighted structural magnetic resonance imaging and genotyped for *MAOA*-uVNTR polymorphism. An automated procedure of FreeSurfer was used to analyze difference in orbitofrontal cortex thickness.

**Results:**

Patients showed a significantly thinner left orbitofrontal cortex (*F*_(1,71)_ = 7.941, *p* = 0.006) and right orbitofrontal cortex (*F*_(1,71)_ = 17.447, *p* < 0.001). For the orbitofrontal cortex sub-region analysis, patients showed a significantly thinner left medial orbitofrontal cortex (*F*_(1,71)_ = 8.117, *p* = 0.006), right medial orbitofrontal cortex (*F*_(1,71)_ = 21.795, *p* < 0.001) and right lateral orbitofrontal cortex (*F*_(1,71)_ = 9.932, *p* = 0.002) compared to healthy controls. No significant interaction of diagnosis and *MAOA*-uVNTR genotype on orbitofrontal cortex thickness was revealed.

**Conclusions:**

Our results suggest that structural alterations of the orbitofrontal cortex may be associated with the pathophysiology of major depressive disorder. Future studies with larger sample sizes are needed to detect a possible association between *MAOA*-uVNTR genotype and orbitofrontal cortex thickness in depression.

## Background

Neuroimaging studies on major depressive disorder (MDD) have consistently identified neuroanatomical alterations in brain regions that participate in affect regulation, such as the prefrontal cortex and limbic system [1]. The prefrontal cortex is known to be a heterogeneous structure both anatomically and functionally and it has been suggested that specific subdivisions of the prefrontal cortex, for instance, the orbitofrontal cortex (OFC), may be differently involved in the pathophysiology of MDD [2]. The OFC is considered to play an important role in mood regulation, and damage to the OFC has been characterized by deficits in emotion and social regulation along with mood lability [3]. Therefore, numerous structural neuroimaging studies have been conducted on the OFC in MDD patients, with studies using whole-brain approaches with voxel-based morphometry reporting gray matter volume reduction of the OFC in MDD patients [4]. In addition, as the OFC itself has been reported to be regionally specialized [5], studies that have separately evaluated both medial and lateral OFC volumes in MDD have reported reduced gray matter volumes in both areas [2]. Studies measuring cortical thickness have reported an association between MDD and cortical thinning of the lateral OFC [6] and medial OFC [7].

Despite these many reports, it is not yet clear what factors may contribute to the alteration of OFC structure in MDD. Genetic polymorphisms that increase the risk for depression are considered to be one of the contributors, as these genetic variants are presumed to act on brain function and architecture [8]. Polymorphisms of the monoamine oxidase A (*MAOA*) gene have been associated with an increased risk of depression [9]. An upstream variable number of tandem repeats (uVNTR) polymorphism in the promoter region of the *MAOA* gene consists of a 30-bp repeat sequence present in 2, 3, 3.5, 4, or 5 repeats (R) [10]. As the 3.5R or 4R alleles were reported to be transcribed 2–10 times more efficiently than 3R or 5R alleles, previous studies have defined *MAOA*-uVNTR polymorphism to produce genotypes with low activity and high activity [11]. Although high-activity *MAOA*-uVNTR variants (*MAOA*-H, genotypes associated with 3.5R or 4R alleles) have been associated with MDD [12], some studies have failed to find such an association [13], and various studies have reported an association between low-activity *MAOA*-uVNTR variants (*MAOA*-L, genotypes associated with 3R or 5R alleles) and MDD [9].

Monoamine oxidase A-upstream variable number of tandem repeats polymorphism has also been associated with structural alterations of brain regions involved in mood regulation [14]. How *MAOA* modulates brain structure has previously been explained by the role of MAOA enzyme in regulating the metabolism of monoamine neurotransmitters [15]. Therefore, numerous imaging genetics studies have been conducted on *MAOA*-uVNTR polymorphism, with a majority reporting *MAOA*-uVNTR genotype to be relevant to OFC structure in healthy controls (HCs). Decreased OFC activity during perceptual matching of angry and fearful faces in healthy individuals with *MAOA*-L, along with increased OFC volume in male *MAOA*-L subjects, has previously been reported [16]. Reduction of OFC volume in *MAOA*-H HCs was also reported [17], whereas *MAOA*-H subjects were shown to have the highest OFC thickness in another study [18].

Cortical thickness refers to the grey matter of the cortex, and shrinkage of neurons, reduction of synaptic spines and lower number of synapses may account for the reduction in grey matter, which may lead to the decrease in function of the thinned area [19]. Whereas measurement of volume may give insufficient information about the dimensions of structure, measurement of thickness enables a continuous measurement across the cortical surface [20], and might be more sensitive to detect structural abnormalities [21]. It has also been suggested that cortical thickness measurements should be preferred over gray matter volume measurements for imaging genetics studies [22]. FreeSurfer has been reported as a highly reliable method for automated cortical thickness measurement [23].

Therefore, we hypothesized MDD patients to exhibit a reduction in OFC thickness compared to HCs. We also hypothesized *MAOA*-uVNTR polymorphism to have influence on the alteration of OFC thickness in MDD, which will be observed as a significant interaction of diagnosis (MDD patients, HCs) and *MAOA*-uVNTR genotype (*MAOA*-H, *MAOA*-L) on OFC thickness. The neurotrophic effects of antidepressants, which may reverse neuronal atrophy, have previously been reported [24]. Therefore, only medication-naïve MDD patients, who had never taken psychotropic medications before, were included in this study when comparing OFC thickness. In addition, MDD is known to be more prevalent among females, and as the *MAOA* gene is located on the X chromosome and sex-specific effects of *MAOA* have previously been reported [25], our patient and control groups only consisted of females.

## Methods

### Participants

We studied 31 medication-naïve female patients with MDD and 43 HCs. Patients were recruited from the outpatient psychiatric clinic of Korea University Anam Hospital located in Seoul, Republic of Korea. Diagnosis was determined by a board-certified psychiatrist, according to the Diagnostic and Statistical Manual for Mental Disorders-IV-Text Revision (DSM-IV-TR), using the Korean version of the Structured Clinical Interview for DSM-IV. Severity of depression was measured by the 17-item Hamilton Depression Rating Scale (HRDS) on the day of magnetic resonance imaging (MRI) acquisition. Patients with primary or comorbid psychiatric diagnoses other than MDD were excluded from the study. Patients suffering from serious or unstable medical illness and primary neurological illness were also excluded. Forty-three age, sex, and education level matched HCs were recruited by advertisements from the community. HCs were screened for major psychiatric histories, and none had a psychiatric disorder. The age of subjects in both groups ranged from 23 to 60 years. All subjects were right-handed as revealed by the Edinburgh Handedness Test and were self-identified Koreans with ethnic origin ascertained by confirming the ethnicity of three generations of the patients’ families. The protocol was approved by the institutional review board of Korea University College of Medicine and signed informed consent was obtained from all participants according to the Declaration of Helsinki.

### MRI acquisition

Three-dimensional structural MRI scans were acquired from a 3.0 T Siemens Trio whole-body imaging system (Siemens Medical Systems, Iselin, NJ, USA), using a T1-weighted magnetization-prepared rapid gradient-echo (MP-RAGE 1900 ms repetition time, 2.6 ms echo time, 220 mm field of view, 256 × 256 matrix size, 176 coronal slices without gap, 1 × 1 × 1 mm^3^ voxels, 16° flip angle, number of excitations = 1). All scans were inspected for motion artifacts and a neuroradiologist confirmed the absence of gross pathological findings.

### MR scan processing and calculation of cortical thickness

Cortical thickness analyses were performed on the three-dimensional model of cortical surface reconstructions computed from T1 images using the FreeSurfer 5.0 software package (Massachusetts General Hospital, Boston, U.S., http://surfer.nmr.mgh.harvard.edu). The details of technical aspects in these procedures have been described in previous publications [26–30]. Briefly, the implanted processing stream involved motion correction of volumetric T1-weighted images, removal of non-brain tissue using a hybrid watershed/surface deformation procedure, automated Talairach transformation of each subject’s native brain, segmentation of the gray matter–white matter volumetric structures, inflation of cortical surface to an average spherical surface to locate both the pial surface and the gray matter–white matter boundary, intensity normalization, and automated topology correction. Transition of gray/white matter and pial boundary was indicated by detecting the greatest shift in intensity through surface deformation. The cortex of each subject was then visually inspected. The entire cerebral cortex was parcelated into units based on gyral and sulcal structure. The computed cortical thickness was defined as the shortest distance between the pial surface and the gray matter–white matter boundary at each given point across the cortex. The cortical maps were generated by computing mean cortical thickness for each subject at each vertex, right and left hemispheres separately, and mapping these data to the surface of an average brain template enabling visualization of data across the entire cortical surface. Smoothing with a Gaussian kernel of 10 mm full width at half-maximum was performed on the cortical maps of each subject for the entire cortex analyses.

### Calculation of averaged cortical thickness within region of interest

**Fig. 1:**
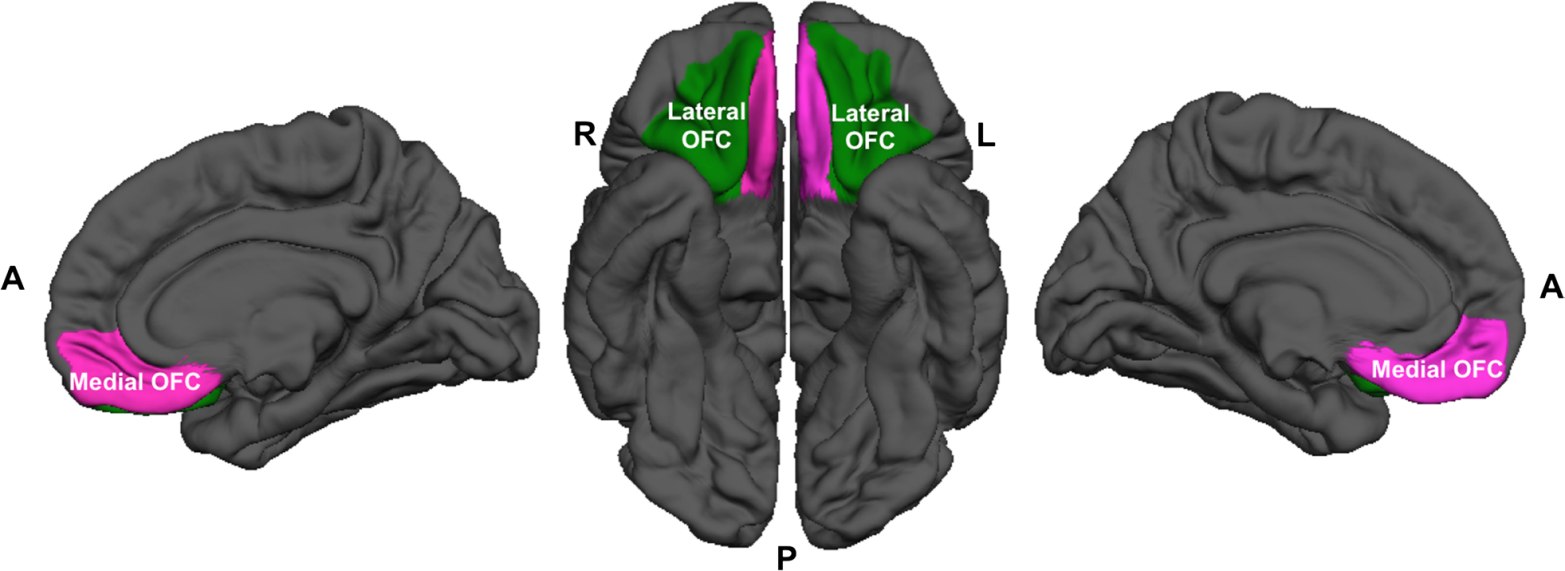
The orbitofrontal cortex including the medial and lateral sub-regions, defined with reference to the Desikan–Killiany atlas

This study focused on the difference in cortical thickness within a region of interest (ROI), the OFC. As the OFC has been reported to be regionally specialized, cortical thickness of the medial and lateral sub-regions of the OFC was also separately calculated [5]. The ROI was defined with reference to the Desikan–Killiany atlas (Fig. 1) [30], and the FreeSurfer package automatically estimated individual cortical thickness of the ROI for the left and right hemisphere by mapping each ROI mask. Cortical thickness measurement of each vertex of the subjects’ surface was mapped on a common spherical coordinate system using a spherical transformation.

### Genetic analyses and identification of subgroups

Patients and HCs were genotyped for *MAOA*-uVNTR polymorphism. Venous blood was drawn from each subject using a protocol approved by the Ethics Committee of the Korea University Medical Center. DNA was isolated using standard techniques. Genotyping of the *MAOA*-uVNTR polymorphism was performed as described by Manor et al. [31]. According to genotype, patients and HCs were divided into two subgroups: *MAOA*-uVNTR high-activity allele carriers (3R/4R, 4R/4R, *MAOA*-H) versus homogenous *MAOA*-uVNTR low-activity allele carriers (2R/3R, 3R/3R, *MAOA*-L). Although heterozygous females with one or two low-activity alleles have been grouped as *MAOA*-L in some studies, others have grouped such subjects as *MAOA*-H [9], which is in accordance with our subgrouping.

### Statistical analysis

Differences in demographic and clinical characteristics between MDD patients and HCs were analyzed using one-way ANOVA for continuous variables (age, years of education, illness duration and HDRS scores). Hardy–Weinberg equilibrium (HWE) of allele frequencies of *MAOA*-uVNTR was analyzed using a Chi-square test. The difference in averaged cortical thickness (mm) of the OFC, including the medial and lateral sub-regions, between MDD patients and HCs was analyzed using ANCOVA with age as a covariate. When comparing the OFC as a whole, the right and left OFC were separately tested, at *p* < 0.05/2 = 0.025 for multiple comparisons with Bonferroni correction. When comparing the sub-regions of the OFC, 4 sub-regions (left medial OFC, left lateral OFC, right medial OFC, and right lateral OFC) were separately tested at *p* < 0.05/4 = 0.0125 after Bonferroni correction. The interaction of diagnosis (MDD patients, HCs) and *MAOA*-uVNTR genotype (*MAOA*-H, *MAOA*-L) on OFC thickness was examined using ANCOVA with age as a covariate.

## Results

### Demographic and clinical characteristics

Age, years of education, duration of illness, and HDRS scores of 31 MDD patients and 43 HCs are shown in Table 1. No significant difference in age and years of education was found between MDD patients and HCs. The allele frequencies of *MAOA*-uVNTR in MDD patients and HCs were in agreement with the HWE as shown in Table 2.

**Table 1:** Demographic and clinical characteristics of medication-naïve female patients with MDD and HCs

Demographic data	MDD patients	HCs	F	*P*
N	31	43	–	–
Age, years	40.83 (9.69)	43.51 (12.21)	1.020	0.316
Illness duration, months	8.51 (20.02)	–	–	–
Education, years	12.41 (3.29)	13.88 (3.23)	3.640	0.060
HDRS-17	20.96 (5.09)	2.41 (2.20)	453.768	<0.001*

All data are given as mean (standard deviation)

*MDD* major depressive disorder, *HCs* healthy controls, HDRS-17 17-item Hamilton Depression Rating Scale

* Significant *p* value; *p* < 0.05

**Table 2:** Hardy–Weinberg equilibrium of allele frequencies and genotype distribution of *MAOA*-uVNTR

	Hardy–Weinberg equilibrium of allele frequencies				Genotype distribution, N (%)			
	2R	3R	4R	P	2R/3R	3R/3R	3R/4R	4R/4R
Total	2 (1.35 %)	97 (65.54 %)	49 (33.11 %)	0.670	2 (2.70 %)	30 (40.54 %)	35 (47.29 %)	7 (9.45 %)
MDD patients	2 (3.23 %)	39 (62.9 %)	21 (33.87 %)	0.542	2 (6.45 %)	13 (41.93 %)	11 (35.48 %)	5 (16.12 %)
HCs	0 (0.00 %)	58 (67.44 %)	28 (32.56 %)	0.075	0 (0.00 %)	17 (39.53 %)	24 (55.81 %)	2 (4.65 %)

*MAOA*-*uVNTR* monoamine oxidase A-upstream variable number of tandem repeats, *MDD* major depressive disorder, *HCs* healthy controls

### Cortical thickness analysis

**Fig. 2:**
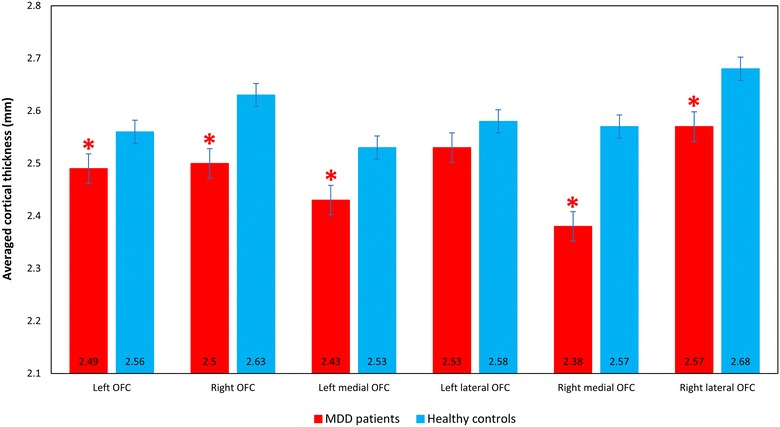
Averaged cortical thickness of the orbitofrontal cortex (OFC) in major depressive disorder (MDD) patients and healthy controls (HCs), as automatically parcelated by FreeSurfer. The *red bars* represent the average cortical thickness of MDD patients in each OFC area. The *blue bars* represent the average cortical thickness of HCs in each OFC area. MDD patients show a significantly thinner cortex in the left OFC, right OFC, left medial OFC, right medial OFC and right lateral OFC compared to HCs

MDD patients showed a significantly (corrected for multiple comparisons with Bonferroni correction) thinner cortex in the left OFC (*F*_(1,71)_ = 7.941, *p* = 0.006; Table 3, Fig. 2), and right OFC (*F*_(1,71)_ = 17.447, *p* < 0.001; Table 3, Fig. 2). For the sub-region analysis, MDD patients showed a significantly (corrected for multiple comparisons with Bonferroni correction) thinner cortex in the left medial OFC (*F*_(1,71)_ = 8.117, *p* = 0.006; Table 3, Fig. 2), right medial OFC (*F*_(1,71)_ = 21.795, *p* < 0.001; Table 3, Fig. 2) and right lateral OFC (*F*_(1,71)_ = 9.932, *p* = 0.002; Table 3, Fig. 2) compared to HCs. No significant interaction of diagnosis (MDD patients, HCs) and *MAOA*-uVNTR genotype (*MAOA*-H, *MAOA*-L) on OFC cortical thickness was revealed for any of the regions (left OFC (*F*_(1,69)_ = 0.345, *p* = 0.559), right OFC (*F*_(1,69)_ = 0.010, *p* = 0.920), left medial OFC (*F*_(1,69)_ = 1.544, *p* = 0.218), right medial OFC (*F*_(1,69)_ = 0.509, *p* = 0.478), left lateral OFC (*F*_(1,69)_ = 0.020, *p* = 0.889), right lateral OFC (*F*_(1,69)_ = 0.063, *p* = 0.802); Table 4). Additionally, the interaction of diagnosis and *MAOA*-uVNTR genotype was examined on the whole brain both regionally and vertex by vertex. For the regional analysis, 34 cortical ROIs with reference to the Desikan–Killiany atlas were analyzed using ANCOVA with age as a covariate. No significant interaction was revealed after Bonferroni correction. For the vertex by vertex analysis, no significant interaction was observed after Monte Carlo Null-Z Simulation (1.3 threshold, bidirectional) in both hemispheres.

**Table 3:** Averaged cortical thickness (mm) of the OFC in MDD patients and HCs

	Cortical thickness		F	*P*
	MDD patients	HCs		
Left OFC	2.49 (0.14)	2.56 (0.16)	7.941	0.006*
Right OFC	2.50 (0.14)	2.63 (0.17)	17.447	<0.001*
Left medial OFC	2.43 (0.13)	2.53 (0.20)	8.117	0.006**
Left lateral OFC	2.53 (0.16)	2.58 (0.17)	3.971	0.050
Right medial OFC	2.38 (0.17)	2.57 (0.20)	21.795	<0.001**
Right lateral OFC	2.57 (0.14)	2.68 (0.19)	9.932	0.002**

ANCOVA analysis with age as a covariate. All data are given as mean (standard deviation)

*OFC* orbitofrontal cortex, *MDD* major depressive disorder, *HCs* healthy controls

* Significance level was corrected for multiple comparisons using Bonferroni correction; *p* < 0.05/2 = 0.025

** Significance level was corrected for multiple comparisons using Bonferroni correction; *p* < 0.05/4 = 0.0125

**Table 4:** Interaction of diagnosis (MDD patients, HCs) and *MAOA*-uVNTR genotype (*MAOA*-H, *MAOA*-L) on OFC cortical thickness

	F	*P*	$\eta^{ 2}_{\text{p}}$	Observed power
Left OFC	0.345	0.559	0.005	0.089
Right OFC	0.010	0.920	< 0.001	0.051
Left medial OFC	1.544	0.218	0.022	0.232
Left lateral OFC	0.020	0.889	<0.001	0.052
Right medial OFC	0.509	0.478	0.007	0.108
Right lateral OFC	0.063	0.802	0.001	0.057

ANCOVA analysis with age as a covariate

*MDD* major depressive disorder, *HCs* healthy controls, *MAOA*-uVNTR monoamine oxidase A-upstream variable number of tandem repeats, *OFC* orbitofrontal cortex

## Discussion

In accordance with our first hypothesis, MDD patients showed a significant reduction in OFC thickness compared to HCs. Our results are similar to previous studies reporting a decrease in OFC thickness in MDD patients [6, 32]. The OFC acts as a key structure in emotion processing and is considered to be important in tasks such as reward-guided behavior, mood regulation, and impulse control [33]. Regional specialization of the OFC has been reported by previous cytoarchitectonic [34], connectivity [5], and functional studies [35], and the medial and lateral parts are assumed to have distinct functions in emotional processing. The lateral OFC is granular [34] and is thought to have evolved from a paleocortical moiety. It is related to medial and dorsal parts of the basal nucleus of the amygdala, as well as to sensory and premotor areas and the posterior cingulate [35]. It is part of the frontostriatal system which is an executive control system, and functionally the lateral OFC has been related to the formation of associations between emotions, especially positive ones, and cognitions [2]. The medial OFC is agranular or dysgranular [34] and is thought to have evolved from an archicortical moiety. It is closely connected to the hippocampal formation, ventrolateral parts of the basal nucleus of the amygdala, dorsolateral prefrontal cortex, and anterior cingulate cortex [2]. Functionally the medial OFC seems to be specialized for emotional processing [35], particularly for negative emotions [34]. Lesions in the OFC have previously been associated with abnormalities in affective behaviors such as depressed mood, anger, affective instability, irritability, and anxiety symptoms [36]. Decreases in cortical thickness, neuronal sizes, and neuronal and glial densities in cortical layers of the OFC in patients with MDD have also been reported [37]. Our results are in accordance with such previous studies reporting structural alterations of the OFC to be a part of the foundation that underlies several clinical features of MDD [2].

Most neuroimaging studies on MDD have been volume based, and relatively fewer studies have investigated the change in cortical thickness of MDD patients compared to HCs [6]. In addition, although various imaging genetics studies have reported changes in cortical thickness of brain areas influenced by certain genetic polymorphisms, most studies were carried out on HCs and not on MDD patients [18]. Our study is meaningful in that we attempted to investigate both the difference in cortical thickness of the OFC between medication-naïve MDD patients and HCs, and the interaction of diagnosis and genotype on OFC thickness. However, despite the fact that previous studies on healthy controls have reported a decrease in lateral OFC thickness [17] and an increase in OFC volume in *MAOA*-L individuals [16], we did not detect a significant association between *MAOA*-uVNTR polymorphism and OFC thickness in MDD.

Considered a limitation, we relied on a relatively small sample size, which may have influenced our negative finding on diagnosis and genotype interaction on OFC thickness. While our sample size was similar to those of recent neuroimaging studies, future studies including a larger sample size may be helpful in demonstrating more confident results. A recent study on sample size estimation of cortical thickness analysis suggested that 50 subjects in each group are required to reliably detect cortical thickness changes of 0.25 mm over 95 % of the entire cortical surface with surface-based smoothing of 10-mm FWHM [38]. However, there is still no consensus on sample size estimation in analyses derived from cortical modeling procedure performed by FreeSurfer. In addition, although we purposely included only female subjects for reasons previously stated, the fact that all subjects were females may be considered as a limitation in the context of generalizing our findings. The potential neuroprotective effect of estrogen has been suggested by previous studies [39], and *MAOA* genotype-dependent structural changes in the OFC have previously been observed in males but not females [16, 17]. Moreover, studies on *MAOA* genotype variation have persistently reported a greater vulnerability of men to the effects of the *MAOA*-L on brain structure, function and connectivity. However, as males with MDD have been reported to have more pronounced neuroanatomic alterations compared to females with MDD, and this in turn could be interpreted as males needing a more pronounced brain structural alteration to develop depressive symptoms [2], our results could be considered even more meaningful as we observed cortical thickness change in subjects composed only of females. Furthermore, whereas males are hemizygous carriers of either one *MAOA* allele, females are heterozygotes. As *MAOA*-uVNTR polymorphism maps to an X chromosome region suspected to escape the normal X chromosome inactivation [40], it is difficult to compare heterozygous females in terms of enzymatic activity. However, similar to our study, previous studies have divided females into two main groups based on their *MAOA*-uVNTR genotype: *MAOA*-L (subjects homozygous for the shortest uVNTR allele) and *MAOA*-H (subjects homozygous for long uVNTR alleles, together with subjects heterozygous for a long and the shortest allele) [9]. Finally, the use of only one locus of the *MAOA* gene may be considered a limitation, and a validation study using an independent case–control cohort may be needed to solidify the conclusion drawn from our results. Previous imaging genetics studies have investigated numerous single-nucleotide polymorphisms of a certain gene and their association with brain structure [41]. Future studies which investigate comprehensively the association between various genetic variations of *MAOA* and structural alterations of the OFC will help shed light on the influence the *MAOA* gene has on OFC structure.

## Conclusions

In conclusion, although the OFC is not the only brain region implicated in depression, our results do suggest that structural alterations of the OFC may be associated with the pathophysiology of MDD. Future studies with larger sample sizes are needed to detect a possible association between *MAOA*-uVNTR genotype and OFC thickness in depression, which will help investigate the mechanistic hypotheses motivated by our results.

## Abbreviations

MAOA-uVNTR: monoamine oxidase A-upstream variable number of tandem repeats
MDD: major depressive disorder
OFC: orbitofrontal cortex
MAOA-H: high-activity monoamine oxidase A-upstream variable number of tandem repeats variant
MAOA-L: low-activity monoamine oxidase A-upstream variable number of tandem repeats variants
HCs: healthy controls
DSM-IV-TR: Diagnostic and Statistical Manual for Mental Disorders-IV-Text Revision
HRDS: Hamilton Depression Rating Scale
MRI: magnetic resonance imaging
ROI: region of interest
